# An Infected Simple Renal Cyst at Each Pole of the Left Kidney and Its Management: A Case Report

**DOI:** 10.7759/cureus.26044

**Published:** 2022-06-17

**Authors:** Tuba Khan, Taha Sajjad, Hasham Masood Qureshi, Ayesha Fonseca, Aadil Khan, Shehar Bano, Uchenna E Ezenagu

**Affiliations:** 1 Medicine and Surgery, Ziauddin University, Clifton, USA; 2 Medical Education, Mountain Vista Medical Center (MVMC), Phoenix, USA; 3 Internal Medicine, Isra University Hospital, Hyderabad, PAK; 4 Medicine, American University of Integrative Sciences (AUIS) School of Medicine, St. Michael, BRB; 5 Internal Medicine, Lala Lajpat Rai (LLR) Hospital, Kanpur, IND; 6 Internal Medicine, University of Health Sciences, Lahore, PAK; 7 Medicine, Sumy State University Ukraine, Sumy, UKR

**Keywords:** anemia, infection, renal failure, unilateral nephrectomy, renal cyst

## Abstract

A simple renal cyst can become infected spontaneously due to various modes like direct cyst penetration during biopsy or surgical exploration, hematogenous spread of infection, and retrograde infection from the urinary tract. Managing such cases becomes challenging due to the risk of rupture of the cyst, causing sequelae of bacteremia and septic shock. Aspiration of cyst along with imaging modality can strengthen the diagnosis. However, nephrectomy coupled with antibacterial administration is an updated therapeutic intervention for an infected simple renal cyst. Our patient presented with a renal cyst at each pole of the left kidney complicated by infection, and after confirming the diagnosis on computed tomography, we performed a right-sided nephrectomy after proper informed consent. The patient responded well to treatment and improved her quality of life.

## Introduction

A simple renal cyst is the most common manifestation of cystic renal disease [1]. Renal cyst is commonly observed in males, with prevalence increasing with age and rarely occurring before 40 years old [2-4]. They are generally found incidentally and can be single or multiple, bilateral, or unilateral. Anatomically they are located mostly in the cortex region but can be observed in the medulla region during the pyelographic investigation. Clinically, they are mostly asymptomatic; however, they can cause impaired renal function and be infected or malignant depending on patient presentation.

Moreover, the infected simple renal cyst is a rare anomaly, and common underlying causes are bloodstream infection, retrograde infection from the urinary tract, or direct exposure to the simple renal cyst during biopsy and operation [5-7]. The most common infective organism is Escherichia coli [8]. Abdominal imaging, likely from computerized tomography (CT) scan and contrast-enhanced ultrasound (CEUS), strengthens the diagnosis, which shows a renal cyst enclosing liquid or semisolid fluid [1,2]. Management of simple renal cysts is improved significantly. Surgical decapsulation of cyst while preserving the adjacent renal tissue is usually adequate. However, it is avoided in poor-risk patients. The histopathological examination of excised cyst is necessary to exclude malignancy. Nephrectomy coupled with antibacterial administration for the culture-positive pathogen is a definite treatment for the infected renal cyst. Herein, we present a case of a 28-year-old female with an infected simple renal cyst at each pole of the left kidney.

## Case presentation

A 28-year-old female came to the emergency department with a chief complaint of abdominal pain associated with nausea, vomiting, fever, and generalized weakness for the past month. The pain was intermittent, dull in nature, radiating downward, relieved by pain killers associated with intermittent and high-grade fever, and multiple episodes of vomiting. No pallor, icterus, cyanosis, clubbing, and lymphadenopathy were found on physical examination, and systemic examination was unremarkable except for mild tenderness in the right flank region. The initial blood investigations showed leucocytosis and anemia (Tables 1, 2). She reported no history of diabetes, hypertension, and tuberculosis.

**Table 1:** Biochemical parameter of the patient. AST: Aspartate transaminase, SGPT: Serum glutamic pyruvic transaminase.

Parameter	Reference Range	Lab Value.
Serum Sodium, mmol/l	137-150	143.6
Serum Potassium, mmol/l	3.5-5.3	4.76
Serum Ionic Calcium, mg/dl	1-5.5	4.2
Serum Bilirubin, Total, mg/dl	0-1.2	0.6
Serum Bilirubin, Direct, mg/dl	0-0.2	0.3
Serum Bilirubin, Indirect, mg/dl	0.2-0.7	0.5
Serum Proteins, g/dl	6.0-8.3	6.1
AST, IU/L	< 40	20
SGPT, IU/L	< 34	12
Serum Alkaline Phosphate, IU/L	< 240	93

**Table 2:** Hematological parameter of the patient. RDWR: Red cell distribution width.

Parameter	Reference Range	Lab value
Haemoglobin, g/dl	12 - 6.5	10.5
Lymphocytes, %	20 - 40	8
Segmented Neutrophils %	40 - 80	85
Mean Cell Volume (MCV), fl	80 - 100	81.1
Mean Corpus Haemoglobin (MCH), pg	27 - 32	31.6
Mean Corpus Hb Concentration (MCHC), g/dl	32 - 35	38.9
RDWR	11.5 - 14.5	18.6
Platelet Count, Lac cells/mm^3^	1.5 - 4.5	1.28
Total Leucocyte Count (TLC), /mm^3^	4000 - 10000	18800
Abs. Neutrophils, /mm^3^	1.2 - 8	17.2 * 103
Abs. Lymphocytes, /mm^3^	0.5 - 5.0	1 * 103
Abs. Eosinophils, /mm^3^	0.5 - 5.0	0.6 * 103
Abs. Eosinophils, /mm^3^	0.5 - 5.0	0.6 * 103
Packed Cell Volume (PCV), %	36 - 46	25.6

**Figure 1:**
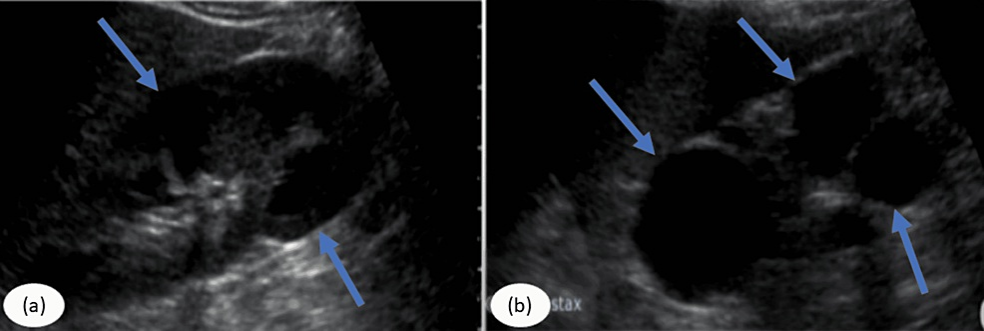
Ultrasound KUB showing cysts of various sizes on each pole of the left kidney (a,b). KUB: kidney, ureter, bladder.

**Figure 2:**
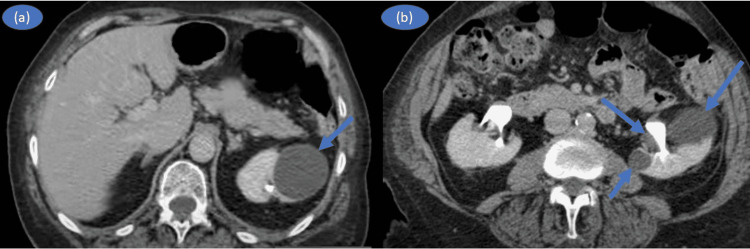
CT abdomen showing cysts at both poles of the left kidney.

Ultrasonography of the whole abdomen was done, which revealed a small-sized left kidney with increased echogenicity, most likely due to three simple renal cysts of size 2x1 cm at the upper, middle, and lower pole respectively (Figures 1a, 1b). Later, these findings were confirmed by a CT scan of the abdomen showing a small, contracted, and structureless left kidney represented as three cystic spaces with thin and regular calcification (Figures 2a, 2b).

**Figure 3:**
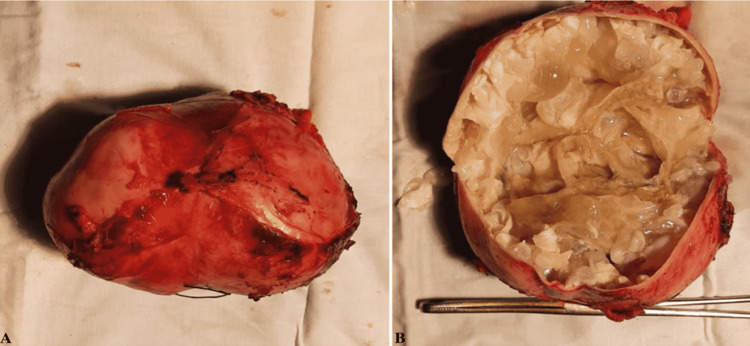
The specimen of excised left kidney.

Owing to the risk of developing chronic kidney disease, operative management was recommended. Written informed consent was taken from the patient, and the patient was prepared for surgery. Laparoscopic left-sided nephrectomy and renal cyst excision were done under general anesthesia. The specimen of the excised left kidney is shown in Figures 3a, 3b. The operation went well without any complications post-operatively, and she was managed with broad-spectrum antibiotics and analgesia. The patient responded well to the treatment. After one week, her condition improved, and she was discharged; however, she was scheduled for a subsequent follow-up.

## Discussion

We reported a 28-year-old female complaining about the abdominal pain associated with constitutional systems, i.e., malaise, fever, nausea, and vomiting. The symptoms persisted for more than a month with temporary relief with medications. Though the biochemical investigations were unremarkable, the complete blood picture showed elevated levels of total leukocytes, predominantly neutrophils, i.e., neutrophilic leucocytosis. Imaging techniques such as ultrasonography and helical computed tomography scan of the abdomen confirmed the existence of three cysts in the patient's left kidney in the upper, middle, and lower pole.

The risk of developing simple renal cysts rises with increasing age. These cysts are more prevalent in males than in females. However, these cysts are more incidental findings because these cysts are asymptomatic frequently until they get infected. Ultrasonography and computed tomography scan are the key radiological techniques for detecting renal cysts. However, ultrasound is less susceptible to identifying small cysts that can increase in size. Therefore, the computed tomography scan is the investigation of choice for detecting renal cysts [8]. Meeks et al. studied the prevalence of renal cysts in patients with sickle cell disease. It was found that more than half of his participants had one or more renal cysts confirmed on the computed tomography scans. In heterozygous sickle cell disease, cases of accelerated renal impairments have been reported due to the increased prevalence of polycystic kidney disease [9]. In polycystic kidney disease, both kidneys are affected by multiple cysts. It occurs due to the overactivation of hypoxia-inducible factors 1 alpha and hypoxia-inducible factor 2 alpha on the epithelial layer and cystic wall, respectively. However, the established sickle cell disease always poses the risk of developing end-stage renal disease. Since the renal medulla is naturally hypoxic and hyperosmolar, this condition promotes the sickling of the red blood cells due to dehydration and vascular occlusion. Recurrent vaso-occlusion and tubular ischemia lead to sickle cell nephropathy progressing to chronic kidney disease, ultimately causing permanent impairments in kidney functions, i.e., end-stage renal disease [10]. 

The renal cysts can easily get infected with the hematogenous route and the urinary tract with retrograde infection. It can also get infected by iatrogenic procedures performed near or at the cyst site. The urinary tract infection can be prevented by proper hygiene and with coverage of appropriate antibiotics. Although nephrectomy is a standard procedure for infected renal cysts, Suwabe et al. studied the transcatheter arterial embolization of the renal artery as a successful procedure for preventing infected renal cysts through the hematogenous route [11]. Transcatheter arterial embolization was preferred over nephrectomy, a less invasive procedure in his research. In this procedure, the blood flow to the infected kidney shuts down, preventing bacterial spread to the cysts through the hematogenous route. However, cases of severe infection after embolization have been reported. In these cases, the embolization was performed in the active stages of infection, restricting the antibiotics from reaching the infection site. Therefore, it is recommended that transcatheter arterial embolization should be performed at least one month after the inflammatory markers become normal. The temperature and inflammatory markers return to normal levels with antibiotics. However, nephrectomy should be preferred over transcatheter arterial embolization if they fail to normalize after a full course of antibiotics [11]. Since the nephrectomy is more invasive and contains a high risk of infection, transcatheter arterial embolization is recommended in elderly patients with multiple co-morbidities.

## Conclusions

A simple renal cyst is a benign condition generally observed during an investigation of other pathological illnesses of kidneys. An infected renal cyst is rare among other manifestations of a simple renal cyst. However, the infected renal cyst is difficult to treat with percutaneous aspiration or surgical exploration. Ultimately nephrectomy coupled with antibacterial administration is the therapeutic choice. Our patient responded well to the treatment without any complications. Further research needs to be done for better therapeutic techniques and improved survival.
